# Phenotypic diversity and distinctiveness of the Belltall garlic landrace

**DOI:** 10.3389/fpls.2022.1004069

**Published:** 2023-01-04

**Authors:** Joan Casals, Ana Rivera, Sonia Campo, Ernest Aymerich, Helena Isern, Daniel Fenero, Alba Garriga, Anna Palou, Amparo Monfort, Werner Howad, Miguel Ángel Rodríguez, Marc Riu, Irma Roig-Villanova

**Affiliations:** ^1^ Miquel Agustí Foundation, Castelldefels, Spain; ^2^ Department of Agri-Food Engineering and Biotechnology, Polytechnic University of Catalonia-BarcelonaTech, Castelldefels, Spain; ^3^ Serra Húnter Fellows, Polytechnic University of Catalonia-BarcelonaTech, Castelldefels, Spain; ^4^ Institut de Recerca i Tecnologia Agroalimentàries (IRTA), Barcelona, Spain; ^5^ Centre for Research in Agricultural Genomics (CRAG), CSIC-IRTA-UAB-UB, Barcelona, Spain; ^6^ Eurecat, Centre Tecnològic de Catalunya, Centre for Omic Sciences (COS), Joint Unit URV-EURECAT, Unique Scientific and Technical Infrastructures (ICTS), Reus, Spain

**Keywords:** *Allium sativum*, projective mapping, genetic diversity, traditional variety, colorimetry

## Abstract

Among the Mediterranean horticultural landraces, garlic is one of the crops most threatened by genetic erosion. Due to its sexual sterility and to the incidence of seed-borne diseases, historical varieties have been widely replaced by commercial cultivars. In Catalonia, despite the historical relevance of the crop, solely the Belltall garlic landrace is cultivated for commercial purposes. To assess the genotypic and phenotypic diversity within the Belltall garlic, we evaluated sixteen local accessions and five recognized traditional and modern varieties as controls. Genetic analysis with SSR and InDel markers showed low genetic diversity within the Belltall population, grouping modern and traditional varieties separately. Farmers and consumers were involved in the definition of the landrace ideotype and classified the materials by means of projective mapping. Scant phenotypic diversity was found within the Belltall landrace, which is characterized by its color profile and the small size of bulb and cloves. The Belltall landrace grown outside its area of origin lost the distinctive quality signals that differentiate the landrace from the commercial cultivars (clove appearance), indicating that the high quality of the landrace is under genotype-by-environment effects (i.e. local adaptation). Moreover, the size of the Belltall sowing clove had a strong effect on the harvested bulb size. Our research represents a case study for the description of the variability within garlic landraces and an approach to quantify the phenomenon of local adaptation that currently drives their conservation.

## Introduction

The Mediterranean basin concentrates different hotspots of secondary diversity of cultivated crops (Ramirez-Villegas et al., 2022). The eastern part of this area was a primary center of domestication for many plant species (Zeder, 2008) and, more notably, the extensive trade networks of dominant civilizations emerged in the region brought exotic crop species that settled and diversified there (Meyer et al., 2012). The crop genetic diversity (either native or imported) was then selected by farmers on the basis of local pedoclimatic conditions and agricultural and gastronomic cultures (Casañas et al., 2017), and resulted in the plethora of landraces that characterize traditional agriculture in the Mediterranean area (Vetelainen et al., 2009). As stated by Rau et al. (2015), stochastic and demographic processes (mutation, drift, migration) plus farmer and natural selection have been the main drivers of the adaptation of landraces to micro-climates, resulting in the emergence of these differentiated genetic groups.

Sexual reproduction plays a key role in crop evolution and domestication, promoting the gene flow through cross-pollination and the combination of adaptive favorable alleles in individuals. With this regard, garlic (*Allium sativum*) can be considered a singular species because of its sterility (Etoh, 1985; Etoh and Simon, 2002), that constrains to propagate the species asexually. Nevertheless, the vegetative mode of propagation has not hindered its capability to accumulate variability, and a wide phenotypic diversity and environmental-adaptation competence has been described in the species (Volk et al., 2004; Egea et al., 2017; Bhusal et al., 2019). As some authors suggest, part of this diversity may probably come from the cross-pollination between wild relatives at the primary center of domestication of the species (Etoh and Simon, 2002). Also, during clonal multiplication, this species accumulates somatic mutations and presents somaclonal variation. More recently, genetic studies indicate that garlic possesses a complex genome which contains duplications and transposable elements. Gene redundancy, differential gene expression and alternative splicing probably have a strong contribution to the phenotypic diversity that we found nowadays in this species (Egea et al., 2017). Therefore, these genetic factors together with seed exchange between contiguous areas and microbial infections (passed on from generation to generation by asexual propagation) contributed to the diversification of garlic (Pooler and Simon, 1993). This diversity is distributed along geographical and environmental gradients, which are correlated with the maintenance and distinctiveness of garlic ecotypes (Mohammadi et al., 2014; Shaaf et al., 2014). Some of these materials have been preserved in delimited areas becoming appreciated landraces.

Nowadays there is an increasing interest in the recovery of some of these garlic landraces because of their sensory profile or sentimental reasons (Ruiz-Aceituno and Lázaro, 2021). However, this species is suffering a rapid genetic erosion because of the replacement of local landraces by modern varieties (Kamenetsky et al., 2007; Gomes Viana et al., 2021). In Spain, garlic has had an important history of cultivation and a wide genetic diversity can be found in *ex situ* germplasm collections (Egea et al., 2017), while much less is found cultivated *in situ*. Noteworthy, most of the original garlic genetic diversity in Catalonia has been lost from the fields (Casals et al., 2017), and solely one local landrace persists in the market due to its prestige among consumers, who argue that it has a differentiated sensory profile (mild flavor, not spicy). This landrace is named after the locality where it is cultivated (Belltall). Different farmers of the municipality grow the landrace in small plots, in rotation with cereals, and sell the product directly to consumers and restaurants. Each farmer preserves its own “seed”, with low exchange rates between them. They describe some degree of phenotypic variability within the landrace but argue that it has a unique sensory profile which is lost when it is grown outside its area of origin (i.e. the distinctive quality of the landrace is controlled by a “favorable” genotype-by-environment (GxE) interaction). Its geographical confinement and the supposed GxE-linked distinctive quality phenotype make the Belltall landrace an interesting case study to analyze the garlic intra-landrace genetic diversity, still scarcely explored (Jabbes et al., 2012; Bhusal et al., 2019; Polyzos et al., 2019; Barboza et al., 2020), and to dissect the genetic and environmental factors that result in the singular quality profile that consumers appreciate.

In this study we characterize the Belltall garlic landrace (a) assessing its genetic and phenotypic intra-landrace diversity; (b) cultivating and comparing in two localities this landrace with commercial controls (modern and traditional) to describe its distinctiveness; (c) involving farmers in the characterization of the diversity through a participatory strategy using the projective mapping methodology; and (d) comparing the robustness of different classification methods (molecular markers, projective mapping, and qualitative and quantitative descriptors).

## Materials and methods

### Plant materials

**Figure 1:**
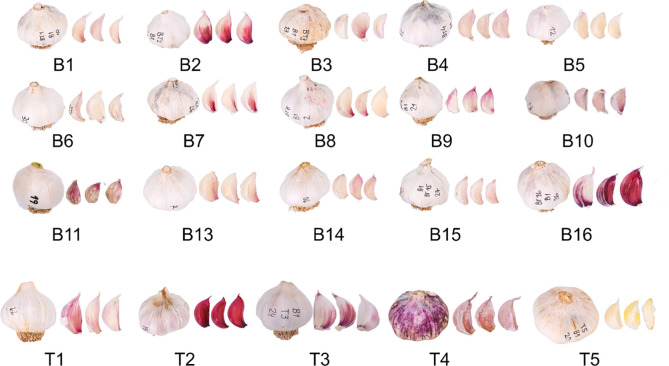
Plant materials used in this study. B1-B16 correspond to the local Belltall garlic populations collected among farmers of the traditional area of cultivation. T1-T5 correspond to the controls. Images show representative garlic bulbs and cloves for each population.

Sixteen accessions of the Belltall garlic landrace were collected among farmers of the traditional area of cultivation (codes B1-B16) the same year of the study, prior to sowing the experimental fields. All the farmers ascribed their populations to the Belltall landrace, describing their “seeds” as historical (more than 50 years of cultivation in the area) (**Figure 1**). Accession B12 showed a very low germination rate in the experimental fields (<20%) and was removed from the study. As controls, we used three renowned landraces from nearby areas: Lautrec garlic from France (T1) and Pedroñete garlic from Spain (T2), recognized with a Protected Geographic Indication (PGI Ail rose de Lautrec and PGI Ajo Morado de Las Pedroñeras, respectively), and Banyoles garlic from Spain (T3). As these landraces have undergone selection processes, their current commercial plant materials show a high degree of homogeneity. Therefore, we only analyzed one accession of each. Two modern varieties widely cultivated in the study area were included as modern controls (T4 and T5).

### Field trials

Plant materials were cultivated in the open field in two localities: the place of origin of the landrace (Belltall, BTLL, UTM: 41°30’26.9”N 1°11’41.2”E, continental Mediterranean climate, altitude 772 a.s.l) and in an experimental station located in Viladecans (Agropolis, AGR, UTM: 41°17’21.5”N 2°02’42.5”E, Mediterranean climate, altitude 0 a.s.l). The two localities represent highly different pedoclimatic conditions: in BTLL the experiment was conducted in a cereal field, with a silty clay loam soil, without irrigation nor fertilization, having prior to sowing high levels of nitrogen (100 mg kg^-1^ N-NO^3−^ dw (dry weight)), phosphorus (60.1 mg kg^-1^ P-Olsen dw), and potassium (409 mg kg^-1^ K_2_O dw); in AGR the experiment was conducted in a horticultural field, with a clay loam soil, with irrigation, having prior to sowing intermediate levels of nitrogen (7 mg kg^-1^ N-NO^3−^ dw), and phosphorus (18.1 mg kg^-1^ P-Olsen dw), and high levels of potassium (434 mg kg^-1^ K_2_O dw). Experiments were sown on 25/11/2020 (BTLL) and 26/11/2020 (AGR). Minimum, maximum and average temperatures during the cropping cycle were: -9.4, 41.1, and 13.9 °C in BTLL, and -1.3, 35, 16.1 °C in AGR. Total precipitation was 271.4 and 191.6 mm in BTLL and AGR, respectively.

In each locality we followed a randomized block design, with 2 blocks and 30 individuals per plot and accession. The individual weight of each clove was recorded before sowing. Cloves were directly sown in the fields, without any prior treatment, following the traditional practices of cultivation in the area. Single rows of plants, separated by 0.7 m, were planted with a distance of 0.2 m between plants. Pests and diseases were chemically controlled. Harvest was done on 13/07/2021 in BTLL and on 14/06/2021 in AGR (230 and 200 days after planting, respectively). After harvest, roots and stalks were removed and bulbs were transported to the laboratory for subsequent analyses.

### Plant genotyping

Genomic DNA was extracted from garlic young leaves using the method of Murray and Thompson (1980) but using mixed alkyltri-methylammoniumbromide (MATAB) as the extraction buffer (0.1 M Tris–HCl pH 8.0, 1.4 M NaCl, 20 mM EDTA, 2% MATAB, 1% PEG 6000, 0.5% sodium sulphite). SSR markers were developed from expressed sequence-tag (EST) *Allium sativum* sequences obtained from GenBank database and analyzed using the SPUTNIK software (Morgante et al., 2002). SSRs with dinucleotide repeats longer than 10 or the equivalent length in nucleotides with tri-, tetra-, or pentanucleotide motifs were considered for primer design using Primer 3 (Rozen and Skaletsky, 2000). Specific primers were designed to amplify 8 markers (**Table 1**) adding an M13 extension (GTAAAACGACGGCCAGT) to the 5’ end of the forward primer. Primer amplifications were first performed under temperature gradient conditions to optimize the amplification. Once the optimal conditions of temperature and Mg concentration were established for each marker, all of them were amplified in a PCR cycler SimpliAmp^™^ (Applied Biosystems), and fragment analysis was performed in an automated sequencer ABI PRISM^®^ 3130*xl* (Applied Biosystems) according to standard protocol and amplification conditions. PCR reaction: PCR buffer 1X, MgCl_2_ 1.5mM, dNTPs 0.2 mM, Forward primer 0.20 μM, M13* Primer 0.20μM, Reverse primer 0.20 μM, BioTaq Polymerase (Bioline) 1U, ADN 40ng and H_2_O (HPLC grade) to a total volume of 10μl. Amplification: 94°C 1 min.; (94°C 15 sec., 60°C 15 sec., 72°C 30 sec.) x 10 cycles; (94°C 15 sec., 50°C 15 sec., 72°C 30 sec.) x 25 cycles; 72°C 5 min.; hold at 12°C.

**Table 1:** Molecular markers used in this study.

Locus	Motif	Primer sequence 5’- 3’ ^a^	Ta (°C)	N_A_	Allele size range (bp) ^b^	PIC
EAS9953	(T)22	f: CAAAAGGAAGCATGGACCAA	60	6	163 - 175	0.28
		r: TACCATCCATCCGAAATGGT				
EAS8947	(tatg)3	f: GTGATTGGACCCGTAGTCGT	60	4	219 - 227	0.45
		r: AGCACATGCAGTGCCAAATA				
EAS3105	(aag)6	f: CCACGAGAGTGGAGGAGAAG	60	3	230 - 254	0.42
		r: TGGCACCGTAACTATGAACG				
EAS6607	(ggttt)3	f: TCGGAGCATGCTTTCTACCT	60	2	146 - 231	0
		r: TTGAGTGGATGATGTCGTTGA				
ASAlli1	(T)12	f: CTCAACTCATCCATGGACTCGTCATCTCT	60	5	206 - 218	0.47
		r: GATCGTACGTTAGATCGATGTGTGC				
ASSTS1	indel	f: TGGACAATGATGAGTACATGTCAGTCGC	60	3	194 - 197	0.27
		r: CAGATAATTTTGATTACAGAGAATTTGCTGTCAACTT				
ASChtn1	indel	f: CAGCAACAGGCTATGCTGTAGC	60	4	240 - 285	0.27
		r: GAATGAGTTTGCAGCTGCTATGAAGG				
ASCHS1	indel	f: GTGAAGCGCTTCATGATGTACCA	60	1	467	0
		r: GGATGCGCTATCCAAAACACCT				

Ta, annealing temperature; N_A_, number of detected alleles; “f” and “r” preceding the primer sequence indicate “forward” and “reverse”, respectively; PIC, polymorphic information content; ^a^: f primer without M13 extension (GTAAAACGACGGCCAGT); ^b^: product size includes M13 extension.

All amplified bands were treated as genetic markers. For each SSR/InDel the different resulting bands were scored in each genotype as a binary variable (0, absent; 1, present) to construct a rectangular matrix. Polymorphic index content (PIC) for each marker was calculated as PIC= 1-∑(p^2^-q^2^), where p and q are the frequencies of the allele presence and absence, respectively.

### Projective mapping

Fifteen days after harvesting, the garlic samples from BTLL were submitted to visual sensory evaluation by two groups of panelists following the methodology of projective mapping (Risvik et al., 1994). The first group (n=15) was composed of farmers or inhabitants of Belltall, who stated that they had consumed the Belltall garlic since childhood (>20 years), therefore considered experienced panelists; the second group (n=9) was composed of consumers who declared having minimal knowledge of the variety (<5 years), therefore unexperienced panelists. Each panelist was provided with a complete set of garlic accessions, each one represented by one bulb and four unpeeled cloves. To conduct the experiment we followed the methodology described in Barcenas et al. (2004). Prior to the analysis, each panelist was briefly introduced to the projective mapping procedure, although no specific direction in the way samples should be evaluated was facilitated. Panelists performed the classification of the varieties in a 2x2 m square drawn on the floor (mapping space). It was underlined that garlic accessions close on the mapping space would be considered similar, whereas samples located on extremes would represent very different garlic varieties. Once completed, each panelist was asked to place a hypothetical sample that represented the ideotype of the landrace in the dissimilarity space. The bi-dimensional data generated by each panelist was transcribed into X-Y matrices by measuring the distance (in cm) of each accession to the origin (0,0).

### Plant phenotyping

Characterization of the accessions was conducted in each locality by means of 38 traits (19 qualitative and 19 quantitative) (**Supplementary Table 1**). Qualitative descriptors were adapted from IPGRI (2001) and UPOV (2001), and were related to vegetative (“Foliage color”, “Foliage attitude”, “Foliage density”, “Leaf waxiness”, “Shape in cross section”), inflorescence (“Ability to produce scape”, “Flowering stem curvature”, “Flowering stem bulblets”, “Internal structure of mature scape”, “Ability to flower”), bulb (“Shape of mature dry bulbs”, “Bulb shape in longitudinal section”, “Bulb shape in cross section”, “Outer skin color of compound bulb”, “Presence of anthocyanins bulb”, “Bulb structure type”), and clove (“Bulb distribution of cloves”, “Bulb external cloves”, “Skin color of the clove”) traits.

Three quantitative traits were measured during the vegetative period in 10 plants per block (“Length of the inflorescence”, “Length of the leaf”, “Width of the leaf”). At harvest, all the bulbs were weighted individually, in order to correlate this trait with the weight of the sown clove. Six bulbs per block were randomly selected to study “Clove weight” (all cloves per bulb were weighed individually, “Bulb weight”), “Number of cloves per bulb”, and “Bulb diameter”. The coefficient of variation (CV) of the single cloves weight per bulb was calculated to estimate “Clove heterogeneity”. On the same 6 bulbs per block, the color profile was studied in the external skin of the bulb (“Bulb external color”, two measures in the equatorial section) and in two peeled and unpeeled cloves per bulb (“Peeled clove color” and “Clove external color”, respectively). Color was measured using a Konica Minolta CR-410 (Minolta, Osaka, Japan) and expressed as L*, a* and b* coordinates from the CIELAB color space. Within locality, the bulbs of each accession were separated into three groups to analyze garlic quality. Soluble solids content (SSC, in °Brix) was evaluated with a pocket digital refractometer (PAL-1, Atago, Tokyo, Japan) after peeling and squeezing the cloves. Bulks of 10 cloves separated in 3 replicates per locality and accession were used to quantify the dry matter content (DM, in %; 72 h, 60 °C).

### Statistical analyses

All the statistical analyses were performed using R v4.0.3 (R Core Team, 2020). For projective mapping, each X-Y distance matrix of each panelist was considered a group; for molecular markers, the markers were grouped in the different SSR/InDel they were derived from. Multiple Factorial Analysis (MFA) was performed on the different datasets to study the similarity/dissimilarity relationships between accessions. Qualitative and quantitative traits were grouped into different categories according to the organ(s) they described (vegetative, inflorescence, bulb, clove). The Principal Components (PC) extracted from the MFA were used to compute Hierarchical Cluster on Principal Components (HCPC), based on Ward distances. The dissimilarity matrices were plotted either using a dendrogram with the “fviz_dend” function or the graph of individuals in Dim1-Dim2 scatterplots using the “fviz_mfa_ind” function. MFA and HCPC analysis were carried out using the “FactoMinerR” (v2.4) (Lê et al., 2008) and “factoextra” (v1.0.7) (Kassambara and Mundt, 2020) packages. The relationships between the different dissimilarity matrices constructed with the different datasets were examined using the Mantel test (Mantel, 1967) with “vegan” package (v2.6.2) (Oksanen et al., 2022), using the Spearman method with 9999 permutations. Univariate analyses on quantitative traits were performed with boxplot graphs and pairwise-comparisons between groups calculated with the Wilcox test, using “ggplot2” (v3.3.5) (Wickham, 2009) and “ggpubr” (v0.4.0.999) (Kassambara, 2020) packages. Finally, for each accession, phenotypic correlations between “seed size” (weight of the clove sown) and “bulb size” (weight of the respective bulb harvested) were calculated using the Pearson coefficient.

## Results

### Genetic diversity of Belltall garlic

**Figure 2:**
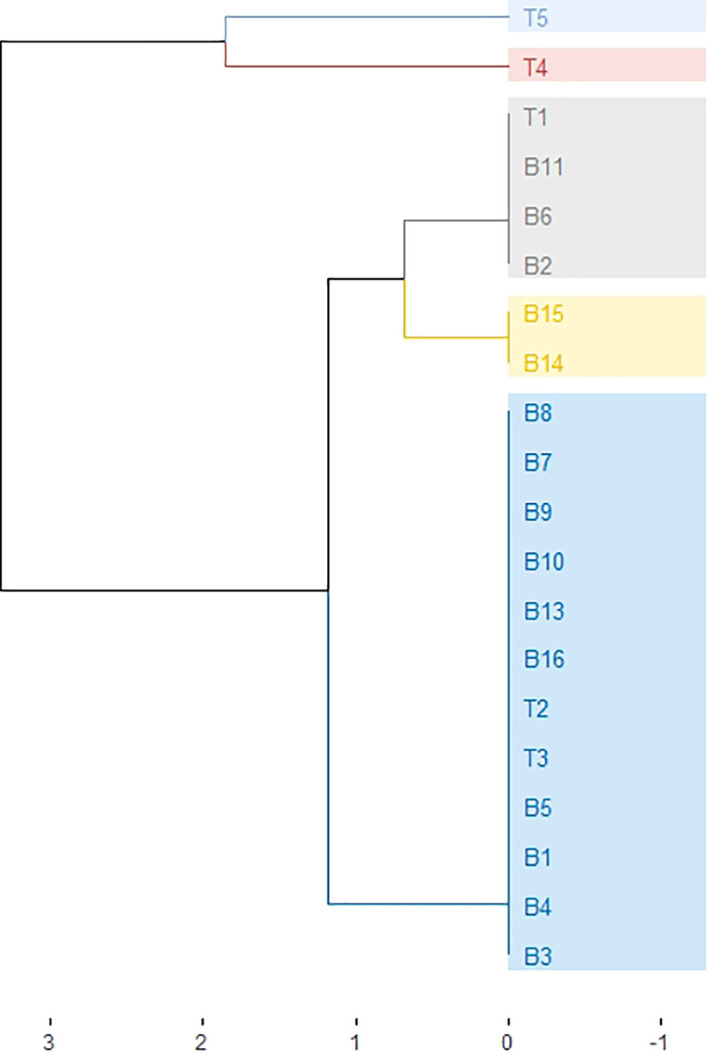
Dissimilarity dendrogram showing the genetic relationships among the Belltall (B) and control (T) garlics. The dendrogram was generated by Hierarchical Cluster on Principal Components (HCPC) analysis based on the eight SSR markers. Accessions with the same genotype are grouped by colors.

The analysis of eight SSR/InDel markers (**Table 1**) in 20 garlic accessions revealed a total of 28 alleles, with a range of 1 to 6 alleles/marker and a mean of 3.5 alleles/marker (**Supplementary Table 2**). In five of the analyzed markers we found more than two alleles in some accessions, which indicates that they amplify more than one locus. Two markers (EAS6607 and ASCHS1) did not reveal any polymorphism in the analyzed genotypes. Among the polymorphic markers, PIC ranged between 0.27 and 0.47, with a mean of 0.36. The most informative markers were ASAli1 (PIC=0.45) and EAS8947 (PIC=0.47) (**Table 1**). The comparison among the Belltall landraces and the controls did not reveal any unique allele in the Belltall populations that can differentiate this landrace from the controls. Based on the HCPC analysis carried out on the binary dissimilarity matrix we constructed a dendrogram to identify the genetic relationships of the Belltall accessions and the controls (**Figure 2**). Modern cultivars (T4, T5) and landraces (B1-B16, T1-T3) were clearly differentiated. Despite few polymorphic markers were found in the Belltall accessions, they grouped in three different clusters. Most of them (10 out of 15) clustered together with the two Spanish landraces used as controls (T2, T3). The remaining five Belltall accessions clustered with the French landrace used as control: T1, B2, B6 and B11 showed the same genotype, while B14 and B15 were polymorphic respect to these for one single marker. Overall, these results show a low genetic diversity among the Belltall accessions, and seem to indicate that within this landrace different genotypes have mixed.

### Classification of Belltall garlic based on visual sensory evaluation (projective mapping)

**Figure 3:**
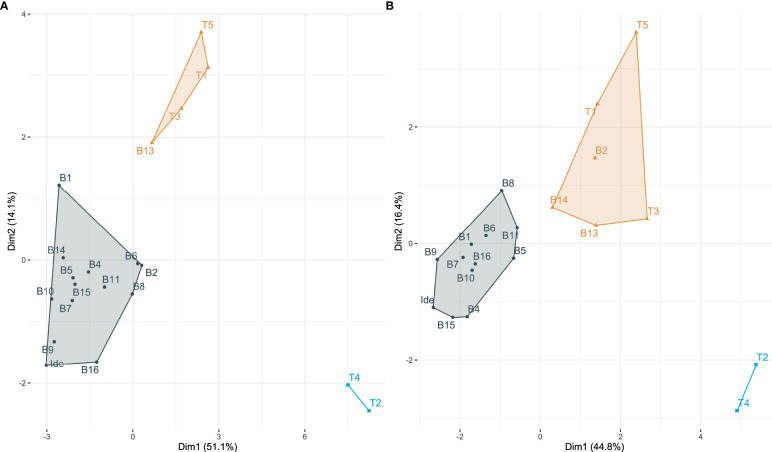
Multiple Factorial Analysis (MFA) plots based on the X-Y dissimilarity distance matrices generated by **(A)** experienced (n=15) and **(B)** unexperienced (n=9) panelists. B1-B16, Belltall traditional accessions; T1-T5, controls; Ide, ideotype of the Belltall landrace. Dim, dimension.

Classification of the experimental materials using the projective mapping methodology was performed by 24 participants, 15 of them experienced with the landrace (> 20 years) and 9 unexperienced (< 5 years) (**Supplementary Figure 1**). MFA conducted on both datasets (**Figure 3**) yielded similar results. The Mantel test between Dim1-Dim2 matrices was highly significant (r=0.817, p<0.0001), revealing that the external phenotype of the landrace is very recognizable and easy to differentiate from other varieties. According to all panelists, accessions were classified in 3 different clusters (**Figure 3**). Experienced panelists described: i) the Belltall cluster, grouping 14 out of the 15 accessions studied, including also the ideotype of Belltall; ii) a cluster grouping the landraces Lautrec (T1) and Banyoles (T3), the modern variety T5 and one Belltall accession (B13); iii) a third cluster, very dissimilar from the rest of the accessions, composed by controls T2 (Pedroñete, landrace) and T4 (modern variety) (**Figure 3A**). The B13 accession was signaled as an outlier of the Belltall cluster by both the experienced and the unexperienced panel; so, this accession was removed from the Belltall group in subsequent analyses. Accessions B9 (both panels), B16 (experienced panel) and B15 (unexperienced panel) were signaled as the closest to the ideotype of the variety.

### Classification of Belltall garlic accessions based on qualitative descriptors

Sixteen out of the 19 qualitative descriptors phenotyped related to vegetative, flowering, bulb, and clove traits showed variation among the accessions (**Supplementary Table 1**). Traits without variation were “Foliage color” (all the accessions were “bluish green”), “Bulb shape in longitudinal section” (all “transverse broad elliptic”), and “Bulb distribution of cloves” (all “radial”). We did not find any single descriptor discriminant between the Belltall landrace and the controls. Also, for six traits we found heterogeneity among the Belltall accessions (“Foliage attitude”, “Foliage density”, “Leaf waxiness”, “Shape of mature dry bulbs”, “Bulb shape in longitudinal section”, and “Presence of anthocyanins bulb”). Overall, the Belltall landrace can be ascribed to a flowering type, without “bulbils on stem”, producing white and circular bulbs, with violet cloves distributed radially and regularly in two sections.

**Figure 4:**
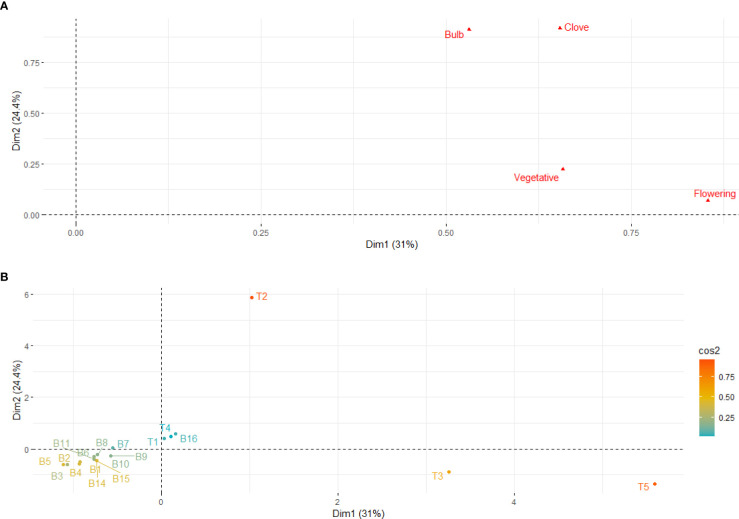
Multiple Factorial Analysis (MFA) of qualitative traits. Dim1-Dim2 scatterplots performed on qualitative variables related to vegetative, flowering, bulb and clove traits. **(A)** contributions of groups of traits to the different dimensions; **(B)** position of the accessions in the Dim1-Dim2 scatterplot. B1-B16, Belltall traditional accessions; T1-T5, controls. Dim, dimension.

To study the similarities between accessions based on the qualitative traits we performed an MFA (**Figure 4**). For this, we classified the variables in four groups related to vegetative, flowering, bulb and clove traits (**Supplementary Table 1**). The two first dimensions (Dim1-Dim2) of the MFA explained 55% of the total variance (**Figure 4A**). We found two groups of variables, with bulb and clove traits closely grouped and plant traits (vegetative and flowering) forming a second group. Thus, bulb and clove characteristics and plant-related traits explain the dissimilarities between accessions differently. The MFA of the 19 accessions analyzed showed that all the Belltall accessions cluster together, with T1 and T4 controls showing a similar profile. The other controls (T2, T3, T5) can be clearly differentiated based on the qualitative descriptors (**Figure 4B**).

### Distinctiveness of the Belltall phenotype based on quantitative traits

**Figure 5:**
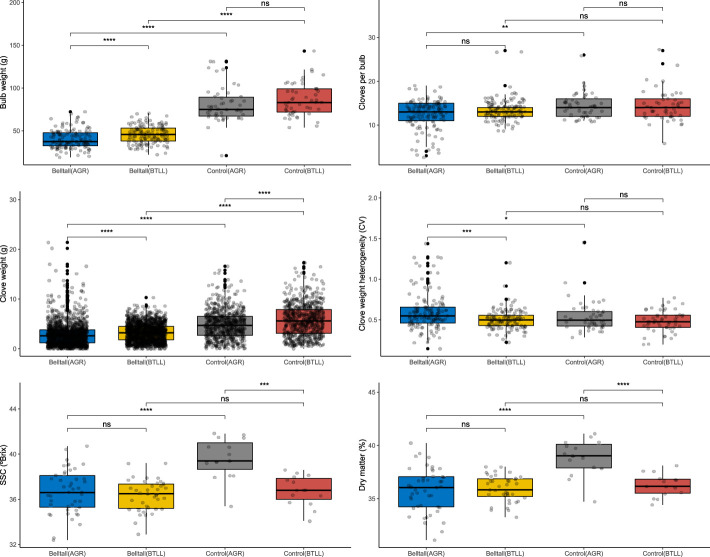
Analysis of quantitative traits of the Belltall landrace in comparison with the controls grown in their original Belltall locality (BTLL) and in a highly different pedoclimatic locality (Agropolis, AGR). Each dot represents a biological replicate. Belltall group includes B1-B11 and B14-B16 accessions, control group includes T1-T5 varieties. Pairwise comparisons between groups were calculated with the Wilcox test. ns (not significant): p > 0.05; *: p <= 0.05; **: p <= 0.01; ***: p <= 0.001; ****: p <= 0.0001.

**Figure 6:**
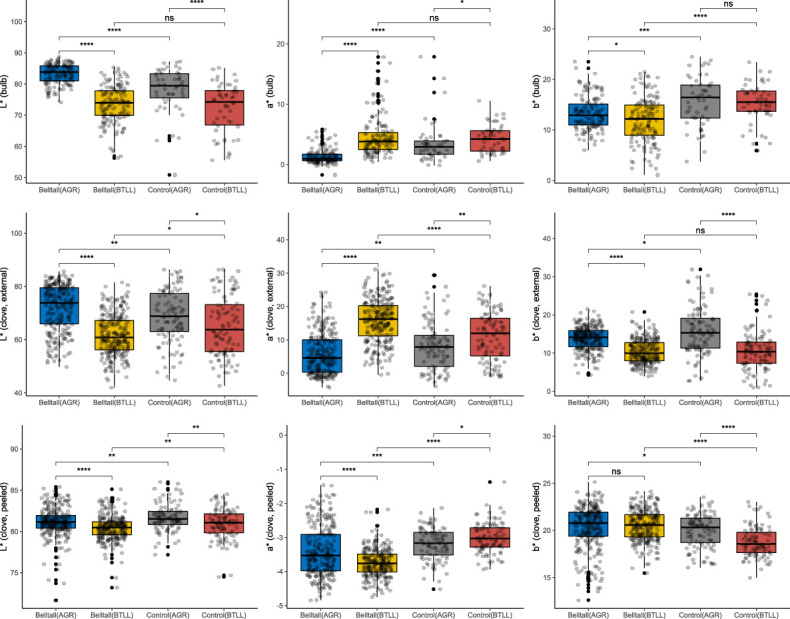
Analysis of the color profile of the Belltall landrace in comparison with the controls grown in their original Belltall locality (BTLL) or in a highly different pedoclimatic locality (Agropolis, AGR). Color coordinates (L*, a*, b*) were obtained on bulbs (external skin), and cloves unpeeled (external skin) and peeled (flesh). Each dot represents a biological replicate. Belltall group includes B1-B11 and B14-B16 accessions, control group includes T1-T5 varieties. Pairwise comparisons between groups were calculated with the Wilcox test. ns (not significant): p > 0.05; *: p <= 0.05; **: p <= 0.01; ***: p <= 0.001; ****: p <= 0.0001.

To further explore the distinctiveness of the Belltall landrace, we analyzed bulb and clove size, quality (SSC, and dry matter) and color profile traits, comparing the phenotype expressed by the Belltall accessions cultivating the plants in their “original locality” (referred as the “Belltall phenotype”) with the controls also grown in Belltall (BTLL) or in a highly different pedoclimatic area (Agropolis, AGR) (**Figures 5**, **6**). Overall, the Belltall phenotype is characterized by a low-size bulb, with bulb and clove weights significantly lower with regard to the controls (**Figure 5**). This small size is not translated into a lower number of cloves per bulb, as the Belltall landrace shows similar values to the controls (13.6 and 14.4 cloves/bulb on average, respectively). When the Belltall landrace was cultivated in AGR, it decreased the size (bulb and clove weight), and significantly increased the clove heterogeneity. Scant differences were found between the Belltall phenotype and the controls regarding sugar content, and dry matter (Belltall, SSC: 36.3 °Brix, DM: 35.8%; controls, SSC: 36.8 °Brix, DM: 36.3%). Moreover, the cultivation of the Belltall landrace in AGR did not alter the SSC and dry matter traits, while in the controls both quality parameters were increased in AGR.

With regard to the color profile, we studied the external color of the bulb and cloves, as well as of the clove flesh (peeled cloves) (**Figure 6**). We found highly significant differences between Belltall and controls regarding all the traits: Belltall landrace showed lower b* values for the external color of the bulb, lower values for L* coordinate and higher values for a* coordinate measured in the cloves, and lower L* and a* values and higher b* values measured in peeled cloves. Additionally, when the Belltall accessions were grown outside of their place of origin, they lost their typical color profile, with significant differences found for all the color variables of Belltall accessions grown in BTLL or AGR.

In summary, the distinctiveness of the Belltall phenotype is related to the low size of the bulbs and cloves, and to the color profile of the bulb (external) and cloves (external and flesh). These traits result from the interaction of the plant material and the growing environment, signaling that positive genotype-by-environment (GxE) interactions are driving its distinctiveness. These results can be useful to promote the cultivation of the local landrace in the Belltall area.

**Figure 7:**
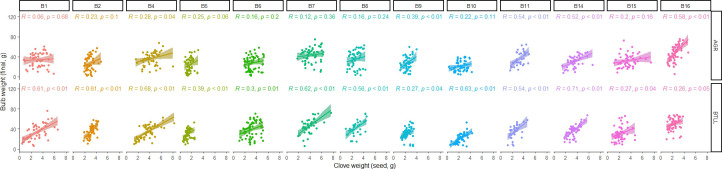
Correlation analysis between the weight of the sowing cloves and their corresponding bulbs weight at harvest of 13 Belltall accessions grown in Agropolis (AGR) and Belltall (BTLL).

To assess the effect of seed size on the final weight of the bulb, we studied the phenotypic correlation between the clove weight of the seed and the weight of their corresponding bulb at harvest in 2081 individuals (**Figure 7**; **Supplementary Figure 2**). We initially performed an analysis grouping all the Belltall accessions on one side and all the controls on the other side. We found a significant and positive correlation for both groups, with higher r values in BTLL (Belltall landrace, r=0.45; controls: r=0.58; p<0.01) than in AGR (Belltall landrace, r=0.31; controls=0.44; p<0.01). For the Belltall landrace, individualized analysis by accessions showed that in BTLL all the correlations were significant (p<0.05) with a range of variation for the r coefficient from 0.26 to 0.71, whereas in AGR solely six out of the 13 accessions showed a significant correlation (range of variation for the r coefficient for the significant correlations: 0.58-0.28). A similar result was obtained for the controls, in some cases with high correlation coefficients (e.g. r=0.84, p<0.01, T4 in BTLL). Considering the relationship between bulb weight and yield we can infer that very low seed clove sizes could have a detrimental effect on the profitability of the landrace, since they result in bulbs of very small size.

### Interaction between qualitative and quantitative traits

**Figure 8:**
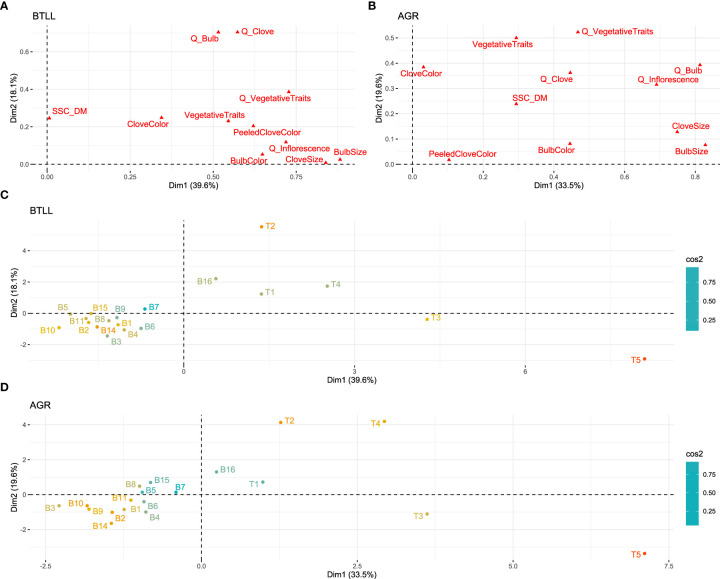
Multiple factorial analysis (MFA) of qualitative and quantitative traits. Dim1-Dim2 scatterplots performed on all the traits measured in the localities of Belltall (BTLL, panels **A, C**), and Agropolis (AGR, panels **B, D**). Traits are grouped by type (qualitative variables are preceded by “Q_”) and categories (vegetative, inflorescence, bulb, clove, chemical composition (SSC_DM), and color (of the bulb, clove and peeled clove)). A and B, contributions of groups of traits to the different dimensions; **(C, D)** positions of the accessions in the Dim1-Dim2 scatterplot. B1-B16, Belltall traditional accessions; T1-T5, controls. Dim, dimension.

We next wanted to test if the Belltall phenotype was distinguishable from the controls when considering all the qualitative and quantitative traits recorded in the accessions. With this aim we performed an MFA considering all the studied traits in both experimental locations (BTLL, **Figures 8A, C**; AGR, **Figures 8B, D**). The Dim1-Dim2 scatterplot, accounting for 57.7% and 53.1% of the total variance in BTLL and AGR fields respectively, showed a clear separation between the Belltall group and the controls. Mantel test revealed strong and significant correspondence between the phenotypic clustering in both localities (r=0.873, p<0.0001). The phenotype of the T1 control (Lautrec garlic) showed the closest relativeness to the Belltall group. The results in BTLL signaled the B16 accession as a possible outlier of the “core landrace”, but this difference was not identified in AGR. Taken altogether, while some aspects of the Belltall singular phenotype are dependent on the growing conditions of the area of origin, its overall distinctiveness is stable across localities. Interestingly, the Mantel test between the dissimilarity matrices constructed based on projective mapping results using the panel of experts (**Figure 3A**) and the standardized descriptors studied in the field of Belltall (**Figure 8C**) resulted in a significant correlation (r=0.652, p=0.0008).

## Discussion

The present study assesses the distinctiveness and intra-varietal diversity of the Belltall garlic landrace based on plant (bulb and clove) traits, SSR/InDel markers and panelists evaluations, as well as the stability of its traditional phenotype when cultivated in a different pedoclimatic area. Although other works dealing with the genetic and phenotypic diversity in garlic landraces did not focus on the intra-landrace diversity (Jabbes et al., 2012; Bhusal et al., 2019; Polyzos et al., 2019; Barboza et al., 2020), it was previously reported that the genetic structure of garlic landraces is correlated to eco-geographical parameters, with geographical and environmental variables acting as important factors in the genetic differentiation between ecotypes (Figliulo et al., 2001; Mohammadi et al., 2014; Shaaf et al., 2014). Accordingly, we have found a low genotypic and phenotypic diversity in the Belltall landrace, either determined by molecular markers, projective mapping or by means of phenotypic descriptors. This could be somehow expected considering the small ecogeographical area where this landrace is cultivated, its unique phenotype according to the farmers descriptions and the absence of sexual reproduction in the species, which limits the introgression of genes from other varieties.

Considering all the phenotypic descriptors, the Belltall landrace is different from closely related garlic landraces such as Lautrec (T1), Pedroñete (T2), or Banyoles (T3), from which it seems to derive. Overall, our results show a possible explanation for the origin of this garlic landrace. According to the molecular results the Belltall landrace could be originated from genotypes from renowned landraces of France (T1, Lautrec garlic) and Spain (T2, Pedroñete garlic; T3, Banyoles garlic), probably due to the exchange of “seeds” with those areas. Alternatively, all these varieties could have originated from a common ancestor. Later, these materials evolved in the specific pedoclimatic conditions of the area, and farmer selection acted on the novel phenotypic variation that appeared in the landrace, boosting its distinctiveness as a differentiated landrace. As described by Pooler and Simon (1993), novel phenotypic variation in garlic can emerge from somatic mutations, as it possibly occurred with accessions B14 and B15 of our study, and also from microbial infections. The latter can affect plant fitness and bulb/clove appearance and be transmitted from generation to generation by asexual propagation (Silander, 1985). Both somatic mutations and microbial infections might have acted as the driving forces of the phenotypic differentiation of the Belltall traditional variety with respect to the original materials.

The main traits that singularize the Belltall phenotype, and that seem to drive consumer loyalty, are the small size (bulb and clove weights) and its specific color profile, affecting the external appearance of the bulb and cloves (unpeeled) and the flesh appearance (peeled cloves). The Belltall phenotype is characterized by low lightness (L*), high values for a* in bulb and unpeeled cloves, and lower values for a* measured in peeled cloves. The color of garlic is the first sensory attribute that a consumer observes, and is considered an important sensory indicator with strong associations with texture, taste, appearance, flavor and overall impression (Liu et al., 2019). Pardo et al. (2007) described that consumer preference negatively correlated with lightness of unpeeled cloves, while Liu et al. (2019) described that high a* values of peeled cloves are indicative of soft texture, spicy taste, yellowish appearance, strong garlic flavor and a high overall impression. Altogether, our results sustain the claimed differentiated sensory profile of the Belltall landrace (mild flavor, not spicy), which is surely the main reason for its conservation. Noteworthy, the Belltall landrace grown outside the original conditions (i.e. AGR) lost completely its distinctive color profile (significant differences were found for all the color measures between the Belltall accessions grown in BTLL and AGR), reinforcing that the quality of the landrace is based on GxE interactions. This distinctiveness of the Belltall phenotype seems crucial for the survival of the landrace, as it permits consumers to easily recognize the landrace in the market.

When we assessed the effect of seed clove size on final bulb weight we observed a significant and positive correlation between both parameters both in the Belltall accessions and in the controls. These correlations were moderate (R<0.6), indicating that the final bulb size is a complex trait, and the initial seed clove size contributes partially to its determination. Our results agree with previous reports from Castellanos et al. (2004); Lima et al. (2019), and Stahlschmidt et al. (1997). As stated by Castellanos et al. (2004), bigger seed cloves have greater carbohydrate and mineral reserves, which promote more vigorous growth and lead to bigger bulbs and cloves at harvest. The different correlation coefficients observed in the different accessions of the landrace shall be related to the different seed health statuses of the accessions, as well as to the different fertility levels present in each locality (Lima et al., 2019). Considering that the small bulb size is a distinctive trait of the Belltall variety, these results contribute to the dissect the environmental and management factors that drive to the Belltall phenotype.

Despite that these distinctive traits were highly affected by GxE effects, the germplasm characterization performed in two localities yielded very similar classifications. The robustness of germplasm classification studies depends on the selection of standardized descriptors, which should be variable in the germplasm collection, and must present a high heritability, as normally the accessions are phenotyped outside their area of origin. In the case of garlic, Figliulo et al. (2001) described high heritabilities (H^2^) for most of the quantitative traits used in our study, supporting the good relationship between the results obtained in the two localities. Using tomato (*Solanum lycopersicum*) as a case study, Figàs et al. (2018) evaluated the robustness of a set of morphological descriptors to classify a set of varieties in different environments, and they found that while most of the descriptors showed high H^2^, some of them were highly affected by GxE interaction. Noteworthy, when all the descriptors were used by means of multivariate analysis to classify the accessions, a good overlap between the different experiments was found. Similarly, in our work, despite the significant differences between localities for individual traits, when all the phenotypic dataset was used to classify the germplasm, the effect of the locality was very low. Complementary to this, we have shown that projective mapping can be a useful tool for fast classification of germplasm. Projective mapping is a methodology of sensory analysis developed in the early ‘90s by Risvik et al. (1994) with the aim to describe dissimilarities between samples (Hopfer and Heymann, 2013). This methodology has been widely applied in sensory experiments (Moss and McSweeney, 2022) but, to our knowledge, it is the first time that is used in studies of genetic diversity with agromorphological descriptors. The significant correlation obtained in the Mantel test between the dissimilarity matrices constructed by the expert panel and the standardized descriptors studied in the field of Belltall points to projective mapping as a promising tool for studies of genetic diversity. Moreover, it can be also helpful in the definition of ideotypes, which is normally a difficult step in plant breeding programs. In our case we asked the participants to position the ideotype of the landrace in the bi-dimensional space where they classified the accessions, resulting in the identification of the accessions closest to the ideotype of the landrace.

In summary, while most of the agromorphological traits of Belltall garlic are under strong genetic control, some of the aspects that make Belltall landrace a singular and appreciated garlic, such as color and size, are dependent on the growing conditions of the area of origin. Altogether, the Belltall garlic landrace seems a good candidate to be distinguished with a European geographical quality label (Protected Designation of Origin (PDO) or PGI), in order to promote the conservation of this variety rooted in the territory.

## Data availability statement

The original contributions presented in the study are included in the article/**Supplementary Material**. Further inquiries can be directed to the corresponding authors.

## Author contributions

IR-V, AR, SC, and JC conceived the study. EA, HI, DF, AG, and AP performed the field experiments and phenotyped the materials. SC, AM, and WH performed the molecular analyses. MAR and MR performed the chemical analyses. JC wrote the first draft of the manuscript. AR, SC, and IR-V revised the manuscript. All authors read and approved the submitted version.
