# Supplementary material for: Phenotypic diversity and distinctiveness of the Belltall garlic landrace
Source: Front Plant Sci. 2023 Jan 4;13:1004069. doi: 10.3389/fpls.2022.1004069 (PMC9846090; doi:10.3389/fpls.2022.1004069)
Supplement: Supplementary file 1 [file DataSheet_1.zip › Supplementary Figures and Captions.DOCX]

**Supplementary Figure 1.** X-Y dissimilarity matrices generated by experienced (pink boxes, >20 years) and unexperienced (white boxes, <5 years) panelists with all the accessions of the experiment by using projective mapping methodology. Red symbols correspond to the Belltall accessions (B1-B16), blue correspond to the controls (T1-T5), and the green triangle to the position of the ideotype in the dissimilarity space according to each panelist judgement. Triangles are used to highlight the closest accessions to the ideotype. Panelists are coded with the numbers shown above the boxes.

**Supplementary Figure 2.** Correlation analysis between the weight of the sowing cloves and their corresponding bulbs weight at harvest of the five controls grown in Agropolis (AGR) and Belltall (BTLL).

**Supplementary Table 1.** A, list of qualitative and quantitative descriptors recorded in the trials, with their corresponding scales of measurement and acronyms. B, mean values per locality (Belltall, BTLL; Agropolis, AGR) of the qualitative and quantitative traits measured in the Belltall accessions (B1-B16) and controls (T1-T5).

**Supplementary Table 2.** Presence of the alleles of each molecular marker in the garlic accessions. B1-B16, Belltall traditional accessions; T1-T5, controls. Molecular markers are described in Table 1.


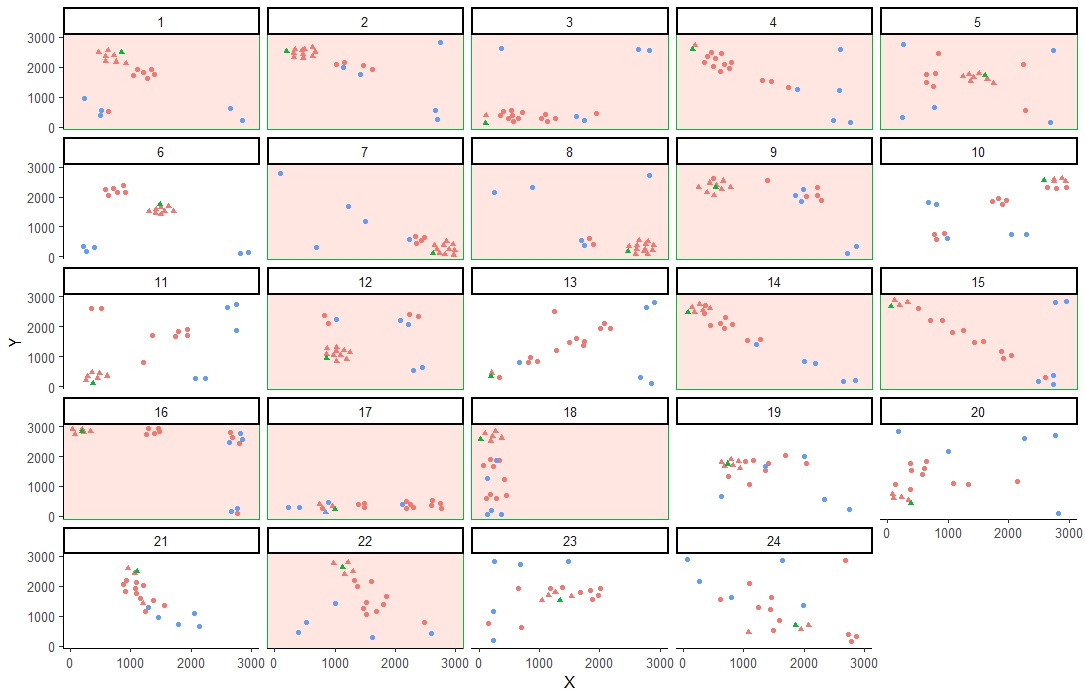


**Supplementary Figure 1.**


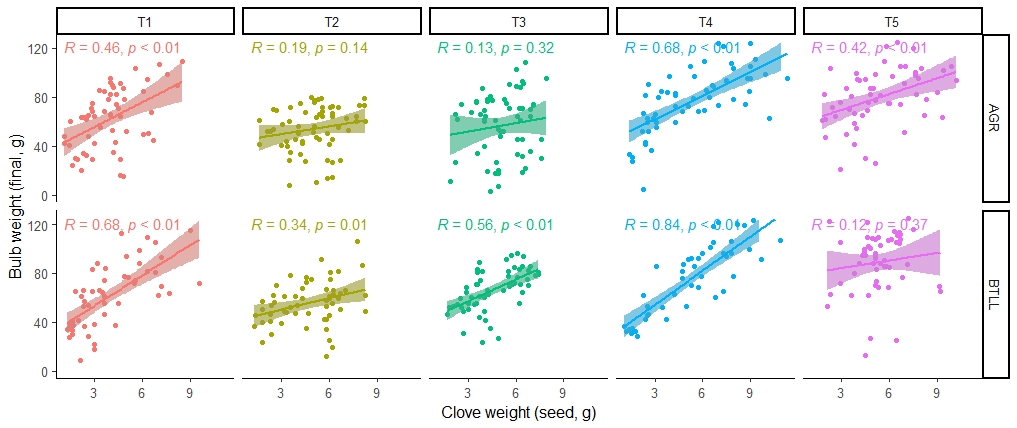


**Supplementary Figure 2.**
